# Noncoding variants are a rare cause of recessive developmental disorders in trans with coding variants

**DOI:** 10.1016/j.gim.2024.101249

**Published:** 2024-09-03

**Authors:** Jenny Lord, Carolina J. Oquendo, Htoo A. Wai, John G. Holloway, Alexandra Martin-Geary, Alexander J.M. Blakes, Elena Arciero, Silvia Domcke, Anne-Marie Childs, Karen Low, Julia Rankin, Genomics England Research Consortium, Diana Baralle, Hilary C. Martin, Nicola Whiffin

**Affiliations:** 1 School of Human Development and Health, Faculty of Medicine, University of Southampton, Southampton, United Kingdom; 2 Sheffield Institute for Translational Neuroscience (SITraN), The University of Sheffield, Sheffield, United Kingdom; 3 Big Data Institute, University of Oxford, United Kingdom; 4 Wellcome Centre for Human Genetics, University of Oxford, United Kingdom; 5 Manchester Centre for Genomic Medicine, Division of Evolution and Genomic Sciences, School of Biological Sciences, Faculty of Biology, Medicine and Health, University of Manchester, Manchester, United Kingdom; 6 Human Genetics Programme, Wellcome Sanger Institute, Wellcome Genome Campus, Hinxton, United Kingdom; 7 Department of Genome Sciences, University of Washington, Seattle, WA; 8 Department of Paediatric Neurology, Leeds teaching Hospitals, United Kingdom; 9 Department of Clinical Genetics, UHBW NHS Trust, Bristol, United Kingdom; 10 Department of Academic Child Health, Bristol Medical School, University of Bristol, Bristol, United Kingdom; 11 Peninsula Clinical Genetics Service, Royal Devon University Hospital, Exeter, United Kingdom; 12 National Institute for Health Research (NIHR) Southampton Biomedical Research Centre, University Hospital Southampton National Health Service (NHS) Foundation Trust, University of Southampton, Southampton, United Kingdom; 13 Program in Medical and Population Genetics, Broad Institute of MIT and Harvard, Cambridge, MA

**Keywords:** Clinical genetic testing, Genomics, Non-coding variants, Rare disorders, Recessive disorders

## Abstract

**Purpose:**

Identifying pathogenic noncoding variants is challenging. A single protein-altering variant is often identified in a recessive gene in individuals with developmental disorders (DD), but the prevalence of pathogenic noncoding “second hits” in *trans* with these is unknown.

**Methods:**

In 4073 genetically undiagnosed rare-disease trio probands from the 100,000 Genomes project, we identified rare heterozygous protein-altering variants in recessive DD-associated genes. We identified rare noncoding variants on the other haplotype in introns, untranslated regions, promoters, and candidate enhancer regions. We clinically evaluated the top candidates for phenotypic fit and performed functional testing where possible.

**Results:**

We identified 3761 rare heterozygous loss-of-function or ClinVar pathogenic variants in recessive DD-associated genes in 2430 probands. For 1366 (36.3%) of these, we identified at least 1 rare noncoding variant in trans. Bioinformatic filtering and clinical review, revealed 7 to be a good clinical fit. After detailed characterization, we identified likely diagnoses for 3 probands (in *GAA, NPHP3*, and *PKHD1*) and candidate diagnoses in a further 3 (*PAH, LAMA2*, and *IGHMBP2)*.

**Conclusion:**

We developed a systematic approach to uncover new diagnoses involving compound heterozygous coding/noncoding variants and conclude that this mechanism is likely to be a rare cause of DDs.

## Introduction

Large-scale exome or genome sequencing of individuals with developmental disorders (DDs) currently identifies a genetic diagnosis for ~30% to 40% of individuals (Wright CF, Campbell P, Eberhardt RY, et al. Optimising diagnostic yield in highly penetrant genomic disease. bioRxiv. Published online July 25, 2022. https://doi.org/10.1101/2022.07.25.22278008).^1^ Analysis in the UK-based Deciphering Developmental Disorders (DDD) study has estimated that, of the ~13,000 participants in that cohort, ~41% are attributable to autosomal de novo coding variants,^2^ ~7% to X-linked coding variants,^3^ and ~3% to autosomal recessive coding variants.^4^ These calculations suggest that even after all DD-associated genes have been identified, a large fraction of individuals with DDs will not be attributable to a Mendelian-acting coding cause and hence remain genetically undiagnosed.

Variants in noncoding regions are increasingly being implicated in DDs. In DDD, it is estimated that ~1% of DDs can be explained by de novo variants in conserved regulatory elements.^5^ In addition, variants in UTRs^6^ and deep intronic regions have been identified as causes of DD.^7,8^ However, the overall contribution of Mendelian-acting noncoding variants to DDs has not been quantified. Sometimes, a single putatively deleterious coding variant is identified in a known recessive gene with a good match to an individual’s phenotype, without an obvious coding “second hit” on the other haplotype. There are examples of noncoding second hits being identified in such individuals, including deep intronic variants in individuals with respiratory disorders (Ellingford JM, Beaman G, Webb K, et al. Genome sequencing enables definitive diagnosis of cystic fibrosis and Primary ciliary Dyskinesia. bioRxiv. October 10, 2018:438838. https://doi.org/10.1101/438838 and Ellingford JM, Thomas HB, Rowlands C, et al. Functional and in-silico interrogation of rare genomic variants impacting RNA splicing for the diagnosis of genomic disorders. bioRxiv. Published online September 26, 2019:781088. https://doi.org/10.1101/781088). However, existing work has not systematically searched for this combined compound heterozygous coding/noncoding mechanism in large rare disease cohorts.

Here, we use genome sequence data from the Genomics England 100,000 Genomes project to investigate the contribution of inherited noncoding regulatory variants in trans with a deleterious coding variant to DDs. We identify individuals with a single loss-of-function or known pathogenic variant in a recessive DD gene, then systematically identify and annotate variants in nearby regulatory regions (including introns, 5′ and 3′ UTRs, promoters, and candidate enhancer regions identified using single-cell-indexed ATAC-seq (sci-ATAC-seq) from fetal brain) in trans that may constitute the “second hit.” We describe clinical follow-up on individuals whose phenotype was a potential fit to the identified gene, followed by transcriptomic investigation on one of them. Overall, we found that this combined compound heterozygous coding/noncoding mechanism explains a very small fraction of DDs but nonetheless accounts for clinically actionable diagnoses.

## Materials and Methods

### Defining the candidate gene set

Genes within which variants are known to cause developmental disorders through a recessive mechanism were identified using the Developmental Disorders Gene to Phenotype (DDG2P) database (downloaded on 02/04/2019) as those with an “allelic requirement” of “biallelic” only (excluding those that also had other inheritance mechanisms), “mutation consequence” including “loss of function,” and “DDD category” of “confirmed” or “probable,” resulting in a set of 793 candidate recessive genes (referred to henceforth as “DDG2P recessive genes”; Supplemental Table 1). We excluded the noncoding RNA gene *RMRP*.

### Identifying individuals with single coding variants

We used the Genomics England (GEL) 100,000 Genomes data set (version 7). We only included probands from the Rare-Disease arm recruited as full trios, comprising an affected proband and both unaffected parents, and that were aligned to GRCh38. We filtered out individuals with variants classified as either tier 1 or tier 2 in the GEL clinical filtering pipeline (https://re-docs.genomicsengland.co.uk/tiering/), which are most likely to have a monogenic diagnosis, plus any individuals with “solved” in their Exit Questionnaire, and probands with subsequently withdrawn consent up to v16 of GEL, leaving 4073 trios. In the remaining individuals, we searched for single heterozygous predicted loss-of-function (pLoF) variants in one of the 793 DDG2P recessive genes, defined based on annotations from Ensembl’s Variant Effect Predictor (v96)^9^ of “stop_gained,” “splice_acceptor,” “splice_donor,” and “frameshift.” pLoFs classified as low confidence by LOFTEEv1.0 were excluded. Additionally, we identified single heterozygous variants in the DDG2P recessive genes that were annotated as pathogenic or likely pathogenic in ClinVar (CLNSIG of “Pathogenic,” “Likely_pathogenic,” or “Pathogenic/Likely_pathogenic”; downloaded on 21/09/21),^10^ with any predicted effect (ie, not limiting to pLoF), and with a review status (CLNREVSTAT) of “criteria_provided,_multiple_submitters,_no_conflicts,” “reviewed_by_expert_panel,” or “practice_guideline.”

Variants were excluded for the following reasons: (1) variant not present in either parent (ie, de novo variants); (2) allele frequency (AF) >0.5% across the 4073 included trios; (3) popmax AF >0.5% gnomAD v2.1.1^11^; (4) <25% or >75% of sequencing reads at that position in the proband contain the variant; (5) VCF “Filter” not “PASS”; and (6) sequencing depth at variant position <6.

In 2 individuals, we identified 2 ClinVar pathogenic/likely pathogenic variants in trans, one of which was protein altering and the other noncoding. These variants were immediately prioritized as candidate diagnoses and put forward for clinical review (see below).

### Defining noncoding regulatory regions

For each of the 793 DDG2P recessive genes, we defined the coordinates of all intronic regions, the 5′ UTR and 3′ UTRs, and a candidate upstream promoter region comprising the first 5000 bps directly upstream of the transcription start site (TSS). Regions were identified for all MANE v1.0 transcripts (MANE Select and Plus Clinical).^12^ Upstream promoter regions were subdivided into a “core promoter,” comprising the first 200 bps upstream, and an “extended promoter” as the remaining region.

Additionally, because most individuals with DD have an abnormality of the nervous system, we used regions of DNA accessible in fetal brain that were identified using sci-ATAC-seq.^13^ We filtered identified peaks to only those identified in ≥5% of cells from fetal cerebrum, or that were in the top 5% of cell-type specificity scores. Peaks were further filtered to only those overlapping the defined upstream promoter region of a DDG2P recessive gene, or that are identified as coaccessible with a region fitting this pro-moter overlap criterion.^13^ These coaccessible regions represent candidate enhancer regions in fetal brain.

Genomic coordinates of all candidate regions are in Supplemental Table 2.

### Identifying candidate noncoding “second hit” variants

For each proband-variant pair (ie, combination of proband and single identified pLoF or ClinVar variant), candidate second hit variants were identified across the noncoding regions defined for the gene containing the coding variant. Only “PASS” variants in the VCF with <25% or >75% of sequencing reads at that position containing the variant were considered. Only heterozygous variants transmitted by the alternative parent to the coding variant (ie, after expected recessive inheritance), with gnomAD v3.0 filtering allele frequency^11^ ≤ 0.5% across all major continental populations, and internal allele frequency in GEL ≤ 0.5% (calculated from the aggregated multisample VCF) were retained. Variants were removed if they overlapped the coding sequence of any MANE transcript (*n = *12) or had a ClinVar annotation of “Benign,” “Likely Benign,” or “Benign/Likely Benign” (*n = *13). This gave 1366 proband-variant pairs with both a single coding variant plus a noncoding variant in trans.

The noncoding variants were prioritized if they matched any of the following region-specific annotations, and the prioritized proband-variant pairs were subsequently subjected to manual clinical review:

- Intronic variants with SpliceAI ≥0.1 (including UTR introns)
- Promoter variants in the “core” region (the first 200 bps directly upstream of the TSS), or that overlap either a sci-ATAC region or a transcription factor binding site annotation from GreenDB^14^ and have CADDv1.6 ≥15
- 5′ UTR variants with SpliceAI ≥0.1, overlapping a transcription factor binding site annotation from GreenDB,^14^ with an annotation from UTRannotator,^15^ or within an internal ribosome entry site (IRES) from IRESbase^16^
- 3′ UTR variants with SpliceAI ≥0.1, overlapping an experimental miRNA binding site collated from 4 studies,^17–20^ or within a polyadenylation signal sequence (defined using Gencode PolyA feature annotation or within a canonical AATAAA motif)
- A variant in any region (including coaccessible sci-ATAC-seq regions) with PhyloP ≥5 and/or CADDv1.6 ≥20

### Clinical filtering

Individuals with candidate noncoding second hit variants were manually reviewed to assess whether the gene was a good clinical match for their phenotype. The individual’s phenotype terms and the disease class under which they were recruited were reviewed by a consultant clinical geneticist (D.B.), against the expected presentation of biallelic pathogenic variants in the relevant genes. Using information on disease presentation from OMIM,^21^ DECIPHER,^22^ and expert knowledge, each proband-gene pair was classified as “probable,” “possible,” or “unlikely.”

In cases which there was a “probable” match between the proband’s recorded phenotype and that associated with the gene, clinical contact forms were submitted to GEL. Clinicians who responded were asked to review the gene as a potential diagnostic candidate for their patient. In cases which a plausible phenotypic match was confirmed, the clinician was invited to offer the patient RNA profiling. In one instance (*NPHP3*, HGNC:7907), the variants had already been recorded as a “partial diagnosis” by the proband’s recruiting center; therefore, contact was not initiated.

Genes were annotated with whether or not they were classified as “Green” in the GEL PanelApp resource^23^ in a gene panel that was applied to the participant, to flag genes that had been considered a priori a possible cause of the participant’s phenotype.

### RNA sequencing and reverse transcription polymerase chain reaction (RT-PCR) functional validations

The participants with variants in *GAA* (HGNC:4065), *LAMA2* (HGNC:6482), and *IGHMBP2* (HGNC:5542) gave informed consent to undergo RNA investigations under the University of Southampton’s Splicing and Disease Study, with ethical approval from the Health Research Authority (IRAS ID 49685, REC 11/SC/0269) and the University of Southampton (ERGO ID 23056). Whole blood samples were collected in PAXgene Blood RNA tubes, and RNA was extracted using the PAXgene Blood RNA kit (Pre-AnalytiX). Random hexamer primers were used to generate complementary DNA via reverse transcription.

For probands with *LAMA2* and *IGHMBP2* variants, RT-PCR was used to assess splicing because of the relatively low expression of those genes in blood (GTEx TPM 0.11 and 8.1), as described in Wai et al^24^ (2022).

For the proband with the *GAA* variants, RNA-seq was undertaken. RNA libraries were prepared by Novogene with rRNA and globin depletion (NEBNext kits), using the NEBNext Ultra Directional RNA Library Prep Kit for Illumina. Sequencing (also by Novogene) was conducted to generate at least 70 million 150-bp paired-end reads on the HiSeq 2000. Raw read quality filtering and adapter trimming were performed by Novogene.

Reads were aligned to the reference genome (GRCh38) using STAR (v2.6.1c) and sequencing reads and sashimi plots in the vicinity of the variants were manually assessed using the Integrative Genomics Viewer (IGV, Broad Institute). rMATS-turbo v4.1.2^25^ was used to detect aberrant splicing, with results filtered to remove events that were outliers in multiple individuals, and OUTRIDER^26^ to test for expression outliers. Twenty-nine additional participants with diverse phenotypes recruited to the Splicing and Disease study and sequenced in the same batch were used as controls. ggsashimi^27^ was used to generate sashimi plots to visualize splicing events. Intron coverage was calculated for all samples in the sequencing batch using HTSeq,^28^ normalized by total gene read count (from STAR) and visualized using ggplot2^29^ in R version 3.5.1.^30^

RNA-seq data generated as part of the 100,000 Genomes Project were available for another proband who carries the *NPHP3* intronic variant ENST00000337331.10:c. 3570+5G>A (NC_000003.12:g.132684549C>T; GRCh38; chr3). At the time of recruitment to the project, blood was collected from a subset of probands in PaxGene tubes. RNA was extracted, globin and ribosomal RNA depleted, and sequencing was conducted by Illumina using 100-bp paired-end reads. Alignment was conducted using Illumina’s DRA-GEN pipeline v3.8.4. IGV^31^ was used to visualize sequencing reads and generate sashimi plots to inspect splicing junctions supported by at least 5 sequencing reads.

## Results

### Identifying and filtering candidate noncoding second hits in genetically undiagnosed rare-disease probands

**Figure 1:**
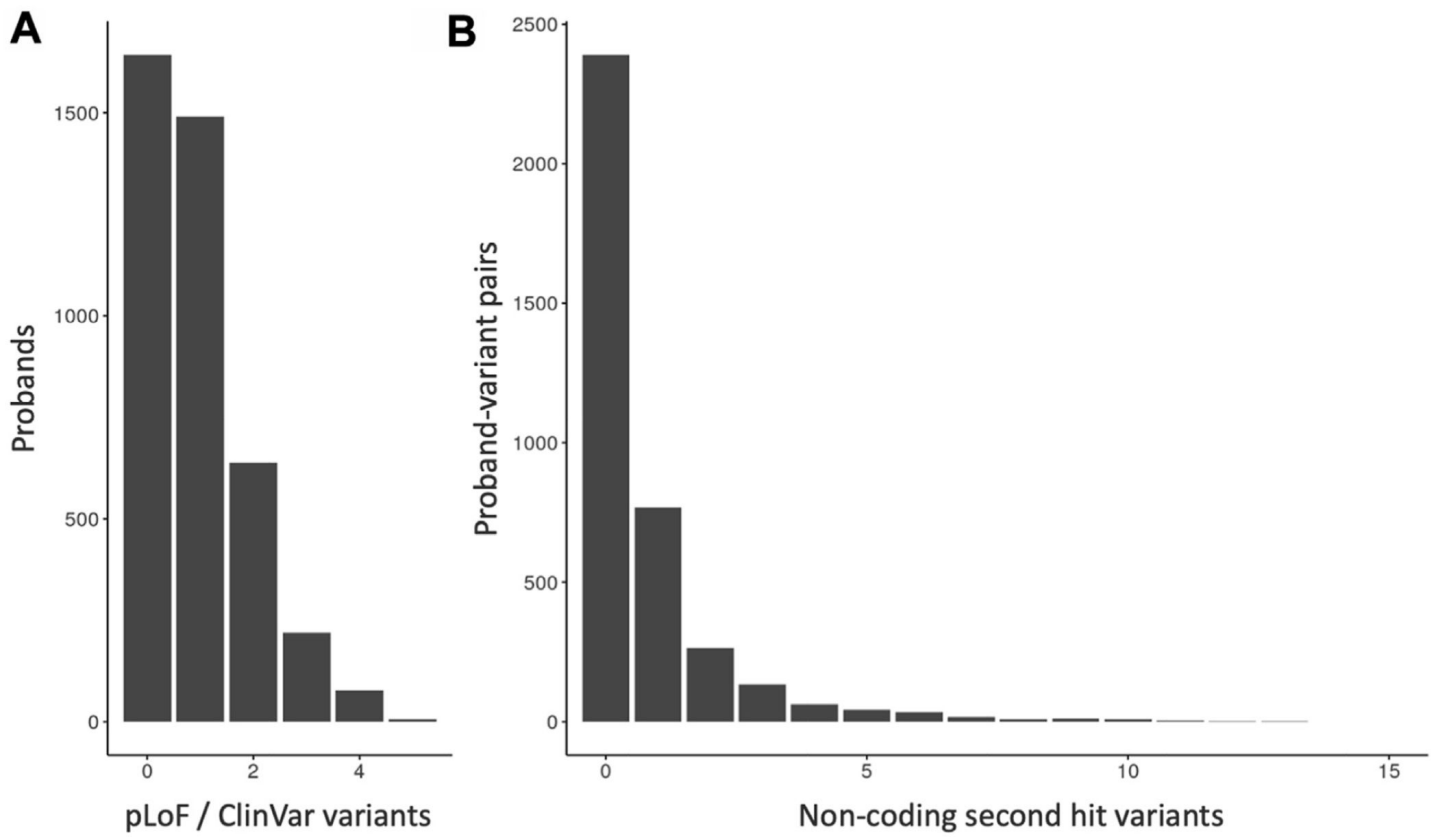
The number of candidate variants identified per proband. A. Bar plot of the number of single pLoF and/or ClinVar (likely) pathogenic variants per individual. B. Bar plot of the number of candidate non-coding second hit variants per proband-variant pair. The *x*-axis is truncated at 15. Four proband-variant pairs had >15 noncoding second hit variants with counts of 16, 20, 25, and 38.

We identified 4073 rare-disease trio probands in GEL without an existing genetic diagnosis. These were recruited for a wide range of primary phenotypes, of which the most common was Neurology and Neurodevelopmental Disorders (1711 probands; Supplemental Figure 1). In 2430 of the 4073 probands (59.7%), we found a single heterozygous pLoF or ClinVar (likely) pathogenic variant in one of 793 DDG2P recessive genes. A total of 940 probands had multiple such variants in different genes (Figure 1A), giving a total of 3761 proband-variant pairs, including 2574 pLoFs and 1187 additional ClinVar variants.

We defined 16,847 distinct noncoding regions associated with our 793 DDG2P recessive genes (Supplemental Table 2), spanning on average 85,937 bps for each gene (range: 5624 to 2,305,299 bps; Supplemental Table 3). For 1366 (36.3%) of our proband-variant pairs, we identified at least 1 rare noncoding variant in trans (ie, inherited from the alternate parent to the coding variant) that passed our quality filters. A total of 597 proband-variant pairs had more than 1 candidate second hit variant (Figure 1B), giving a total of 2973 proband-variant-second-hit combinations. The vast majority of our candidate second hit variants were intronic (2744; 92.3%), reflecting the composition of our noncoding search space.

**Figure 2:**
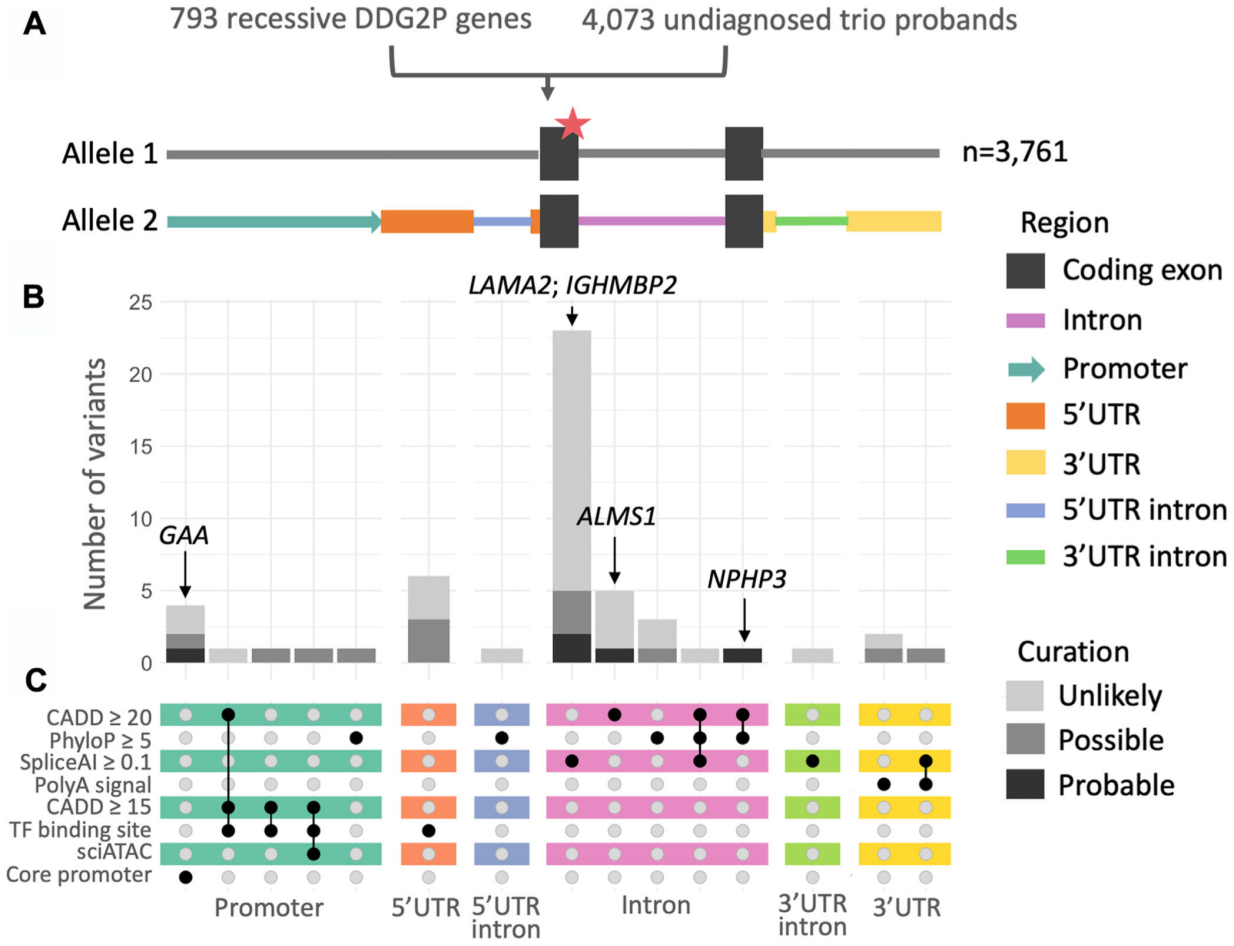
An overview of our approach and the distribution of 52 candidate noncoding second hits across different regions. A. Details of our search focusing on 793 genes with a single pLoF or ClinVar (likely) pathogenic variant in 4073 undiagnosed trio probands. The different candidate noncoding regions analyzed for a second hit are shown in color. B. Count of candidate variants clinically curated as “probable,” “possible,” or “unlikely,” split by region and annotations. The indicated genes are those that were assessed to be a “probable” fit. C. Upset plot of region-specific annotations used to prioritize candidate variants. This plot does not include 2 additional probands who had 2 compound heterozygous ClinVar pathogenic/likely pathogenic variants, 1 protein altering and 1 noncoding.

Given our expectation that most noncoding region variants have a very small, if any, regulatory impact, we created a stringent set of filters to narrow down our list of candidate second hit variants to those that seemed most likely to have an effect (see Materials and Methods). After filtering, we retained 52 candidate second hit variants in 52 probands: 35 intronic, 8 in the promoter region, 6 in the 5′ UTR, and 3′ in the 3 UTR (Figure 2). No candidate variants were identified in candidate distal enhancer regions identified as coaccessible with each promoter region in sci-ATAC-seq data from fetal brain. Additionally, we identified 2 probands with 2 ClinVar pathogenic/likely pathogenic variants in trans, 1 protein altering and 1 noncoding, for a total of 54 candidates.

### Assessing candidate variant match to clinical phenotype

Our 54 candidate coding/noncoding variant pairs were manually reviewed to assess whether they represented a credible explanation for each proband’s phenotype. Seven (13.0%) of the variants were classified as a “probable” match (Table 1), 13 (24.1%) as “possible,” and the remainder as “unlikely.” Of the 7 that were classified as “probable,” all except *NPHP3* were “green genes” on relevant panels that were applied to the proband by GEL^23^ (ie, had been considered a plausible cause a priori), compared with only 12 of the remaining 47 (85.7% vs 25.5%; odds ratio = 16.5; Fisher’s *P =*.004).

One of the “probable” cases, with a frameshift variant and an intronic variant in *ALMS1* 23 bp from a splice donor site, also had a 2-exon duplication in cis with the intronic variant. This had already been considered as a potential 1diagnosis by the recruiting site, with the pLoF variant classified as pathogenic and the duplication as a variant of uncertain significance. Additionally, the intronic variant we detected was present in a homozygous state in 3 members of the UK BioBank (https://decaf.decode.com/region/chr2:73573557-73573567). Taken together, we felt that this meant the intronic variant was unlikely to be pathogenic.

For the remaining 6 probable diagnoses, we attempted to contact the proband’s recruiting clinician through the GEL portal. We were unable to make contact with the clinical team of the individual with the undiagnosed metabolic disorder and the pair of ClinVar pathogenic/likely pathogenic variants in *PAH* (HGNC:8582); therefore, the relevance of these variants remains unclear. We discuss the remaining 5 cases below.

### GAA

We identified a variant in the promoter of *GAA*, 182 bps upstream of the TSS (NG_029761.1:g.69768C>G NC_000017.11:g.80101399C>G; GRCh38; chr17) in trans with a nonsense variant (ENST00000302262.8:c.2577G>A p.(Trp859Ter) g.80118288G>A, GRCh38; chr17) in a proband with a phenotype most similar to limb girdle muscular dystrophy (LGMD). Pathogenic variants in *GAA* cause a glycogen storage disorder called Pompe disease.^32,33^ In silico scores suggest this variant is unlikely to be deleterious (PhyloP < 0; CADD = 5.9), but following contact with the clinical team, biochemical assays confirmed marked deficiency of GAA enzymatic activity in the participant and hence a diagnosis of Pompe disease. Of note, this individual had had GAA enzymatic testing undertaken several years previously and, at that stage, was within normal limits. Across the full GEL cohort (ie, not limited to trios), we observed this variant in a total of 6 probands who also carried a rare missense or pLoF variant. Three of these probands, including our initial case, were reported to have LGMD.

Subsequently, we found that all 3 GEL LGMD probands with the promoter variant also carried a 5′ UTR intronic variant, ENST00000302262.8:c.-32-13T>G (g.80104542 T>G; GRCh38; chr17). This variant is reported as pathogenic in 45 submissions to ClinVar (variation ID:4027), is observed in 36% to 90% of Pompe disease cases (tending to cause later-onset disease), and has been demonstrated to impact splicing, albeit with an incomplete/leaky effect.^34,35^ The promoter and 5′ UTR intronic variants were confirmed to be in cis in our index proband. The 5′ UTR intronic variant was missed in our initial search for second hit variants because of its high frequency in gnomAD (maximum AF 0.0073 in Latinos/Admixed Americans).

**Figure 3:**
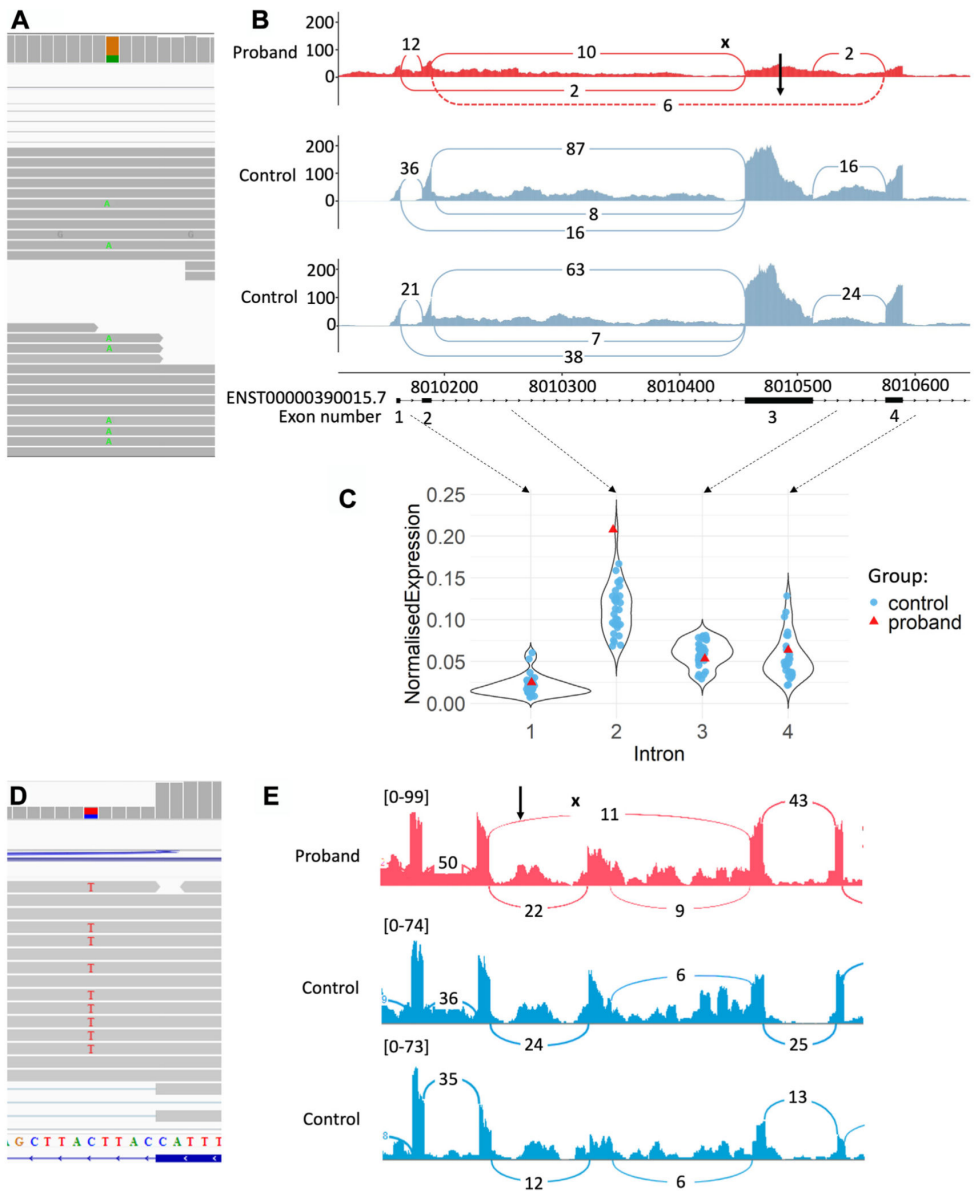
RNA-seq to interrogate candidate variants in *GAA* and *NPHP3*. A. RNAseq reads covering nonsense variant ENST00000302262.8:c.2577G>A (p.Trp859Ter) in *GAA*. Forty-six reads carry the reference allele, whereas 19 carry the alternative allele. B. Sashimi plot showing splicing in the *GAA* proband plus 2 controls from the same sequencing batch. Skipping of exon 3 (ENST0000390015.7) is observed in the proband but not in controls (dashed line, black arrow) due to the variant 13 bp upstream of the exon 3 splice acceptor site (black “x”). C. Normalized expression level per intron for the proband (red) plus 29 controls (gray) sequenced in the same batch. Proband has higher intronic coverage than all controls for intron 2, suggesting that intron retention may be occurring. D. RNAseq reads covering near-splice variant ENST00000337331:c.3570+5G>A (chr3:132684549:C:T) in *NPHP3*, 5 bp from the splice donor site of exon 24, in a different proband identified as a heterozygote with the same noncoding variant as our proband. Six reads carry the reference allele, whereas 9 carry the alternative allele. E. Sashimi plot showing splicing in the individual heterozygous for the ENST00000337331:c.3570+5G>A (chr3:132684549:C:T;NPHP3) variant and 2 control individuals without the variant. Skipping of exon 24 (ENST00000337331) is observed (highlighted by black arrow) in the individual with the variant but not the controls (relative location of variant indicated by black “x”).

Whole-blood-based RNA-seq was conducted on the index proband to assess the impact of the variants on gene expression and splicing. The nonsense variant would be expected to result in nonsense-mediated decay of transcripts from that allele, and although OUTRIDER^26^ did not detect lower expression of the gene relative to other participants in the same sequencing batch, manual inspection of reads in IGV showed a lower proportion of reads carrying the alternate allele (19 vs 46, 29%, Figure 3A). The normal expression level of the gene suggests that the promoter variant does not affect gene expression.

However, we detected altered splicing patterns at the 5′ end of *GAA* near the 5′ UTR intronic variant using rMATS (Supplemental Table 4), showing skipping of exon 3 (“ENST00000390015.7, ENST00000390015.7:c.-32-13T>G Figure 3B), which was not observed in controls and has previously been reported as an outcome of the c.-32-13T>G variant.^36^ This exon contains the start of the *GAA* protein coding sequence and the first 19% of the protein sequence (182 of 952 amino acids). Additionally, we observed a large number of reads mapping to the intronic regions in the area around the 5′ UTR variant, suggesting that there may be full retention of introns, particularly intron 2 (Figure 3C).

Together, these investigations strongly imply that the c.-32-13T>G splice-altering variant is more likely to be the functional non-coding second hit in *GAA* than the promoter variant we initially identified. The combination of this c.-32-13T>G variant in trans with the nonsense variant seems highly likely to be pathogenic in our original index participant.

Although we also identified 2 additional LGMD probands with the c.-32-13T>G variant and a rare missense/pLoF variant in *GAA*, one of these was recruited as a singleton; therefore, the phase of the 2 variants could not be established, and the other was recruited alongside a reportedly affected brother who did not carry the c.-32-13T>G variant. Thus, it remains unclear whether the *GAA* variants are diagnostic in the 2 other LGMD probands.

### NPHP3

In a proband with proteinuric renal disease, we found 2 variants in *NPHP3*, which is known to cause nephronophthisis.^37^ These were a nonsense variant in exon 18 (ENST00000337 331.10:c.2563C>T p.(Gln855Ter) g.132691199G>A; GRCh 38; chr3), reported to be pathogenic in ClinVar with multiple submitters (variation ID:571559), and an intronic variant (ENST00000337331.10:c.3570+5G>A; g.132684549C>T; GRCh38; chr3) 5 bp from the splice donor site of exon 24. Although the SpliceAI score for this variant is below our threshold (0.04), donor +5 variants are known to be under strong selective constraint and often affect splicing.^38,39^ The high CADD (21) and PhloP (6.5) scores for this variant are supportive of a deleterious impact.

This pair of variants had been triaged by GEL’s tiering pipeline and classified as “tier 3” variants and had already been assessed as a potential diagnosis by the proband’s recruiting center. The stop gained variant had been classified as “pathogenic,” and the near-splice variant as “likely pathogenic,” with the overall assessment that the variant pair was partially responsible for the participant’s phenotype. Although RNA from this proband was not available to functionally validate the impact of the near-splice variant, an additional proband with the same intronic variant was identified for which RNA-seq was available, allowing confirmation that this variant (Figure 3D) does, indeed, affect splicing, causing the skipping of exon 24 (Figure 3E). The skipping of this 241-bp exon would disrupt the reading frame and be expected to result in NMD.

### LAMA2

In a proband reported to have congenital myopathy, we found a nonsense variant (ENST00000421865.3:c.3976C>T p.(Arg1326Ter) NC_000006.12:g.129316089C>T; GRCh38; chr6) previously reported to be pathogenic in 8 ClinVar submissions (variation ID:92956), plus an intronic 1-bp duplication (ENST00000421865.3:c.7440-20dup; g.129475 370dup; GRCh38; chr6:129475360:G:GT) upstream of the splice acceptor site of exon 53. SpliceAI predicts a strengthening of the nearby acceptor (0.05) but loss of the donor of the same exon 41 bp away (0.1), predicting an exon skipping impact. Variants that disrupt the canonical acceptor site of exon 53 have previously been reported in individuals with muscular dystrophy (ClinVar variation IDs 1068380 and 954079).^40^

Biallelic variants in *LAMA2* cause muscular dystrophies, with a correlation between phenotype and genotype reported.^41^ Biallelic truncating variants in *LAMA2* are associated with a severe, early onset congenital muscular dystrophy type 1A, whereas missense and some splicing variants lead to a less severe and often later onset LGMD. The proband has a relatively static phenotype with reasonably well-preserved muscle function, which would be consistent with the potentially leaky skipping of the small (12-bp), in-frame exon. The participant’s recruiting clinician confirmed the variant pair as a plausible diagnosis.

Because of the low expected expression of the gene in blood (GTEx TPM = 0.11), RT-PCR rather than RNA-seq was utilized to investigate splicing using RNA from the participant’s blood but proved inconclusive. The expected impact of the variant would be skipping of nearby exon 53, but skipping of this exon was observed in both the individual with the variant and in controls, despite isoform specific expression data from the GTEx portal indicating exon 53 containing ENST00000421865.3 to be the only isoform of the gene with non-zero expression in blood (Supplemental Figure 2). We were unable to assess RNA from a disease relevant tissue (eg, muscle biopsy). Thus, taken together, although these *LAMA2* variants seem very good candidates for causing this participant’s phenotype, we are unable to show this definitively because we could not detect a functional effect of the noncoding variant.

### IGHMBP2

A pair of variants in *IGHMBP2* (HGNC:5542), a gene known to cause Charcot-Marie-Tooth disease (CMT), were found in a proband reported to have CMT disease. We identified a 1-bp deletion in exon 13 (ENST00000255078:c.2429del p.(Pro810LeufsTer21) NC_000011.10:g.68936909del; GRCh 38; chr11:68936904:GC:G), expected to lead to a frameshift in trans with an intronic single-nucleotide variation (ENST00000255078.8:c.1235+450G>A; g.68929807G>A; GRCh38; chr11), 450 bp from the splice donor site of exon 8. The variant introduces a new “AG” motif, with the potential to act as a splice acceptor site (SpliceAI acceptor gain 0.12), which would likely result in the generation of a cryptic exon. This variant has previously been reported in ClinVar as a variant of uncertain significance (variation ID:2430634), with an accompanying comment asserting functional testing of the variant revealed leaky abnormal splicing. However, RT-PCR on blood derived RNA from the proband was unable to confirm this previously reported splicing impact (Supplemental Figure 2). It may be that peripheral neurons would be a more relevant tissue to examine.

Again, there is reported genotype-phenotype correlation for this gene, with the severity of the disorder linked to the nature of the variants. Loss-of-function variants and missense variants in conserved residues in or near the DNA helicase domain are associated with severe, distal hereditary motor neuronopathy (type VI), whereas axonal CMT (type 2S) is associated with less disruptive variants, which allow some residual protein function.^42^ This milder CMT phenotype would be expected if the intronic variant’s impact on splicing is incomplete. Nonetheless, because we were unable to demonstrate a functional impact of this noncoding variant in our patient (despite one having been reported previously in ClinVar), we cannot definitively show that these 2 variants are, in combination, pathogenic; therefore, this case remains inconclusive.

### PKHD1

We identified 2 variants in *PKHD1* (HGNC:9016) in an individual with cystic kidney disease—a missense variant in exon 30 (ENST00000371117.8:c.3467C>T p.(Ser1156Leu) g.52028249G>A; GRCh38; chr6) and a deep intronic splicing variant in intron 46, 653 bp from the nearest splice site (ENST00000371117.8:c.7350+653A>G g.51882440T>C; GRCh38; chr6), both of which are classified as pathogenic/likely pathogenic in ClinVar (ClinVar variant IDs 636580 and 551996, respectively). The splicing variant generates a new cryptic splice donor site within the intron, leading to the inclusion of a 116-bp cryptic exon and the introduction of a premature termination codon.^43^ The variant pair had already been identified and reported to the individual’s clinical team, who confirmed this as a diagnosis for their condition.

## Discussion

Here, we systematically identified, annotated, and prioritized noncoding variants in trans with high-impact or known pathogenic coding variants, across a large cohort of undiagnosed individuals with rare disease. We successfully identified likely diagnoses for 3 probands, each with unique variant combinations in distinct genes (*GAA, NPHP3*, and *PKHD1*). For 3 further probands, we identified candidate diagnoses (in *PAH, LAMA2*, and *IGHMBP2*) but did not have sufficient evidence to categorize them as likely diagnostic.

A key conclusion of this work is that the proposed mechanism of a noncoding second hit in combination with a single heterozygous coding variant is unlikely to explain a large fraction of undiagnosed DD cases. This is despite a large proportion (~60%) having a single pLoF in a known recessive DD gene. Nevertheless, there are some key reasons why our reported diagnostic rate is likely an underestimate. First, we did not perform an upstream clinical review because of the broad spectrum of DDs and incompleteness of phenotypic data in GEL but rather included all rare-disease probands without a complete existing diagnosis. Hence, not all of the 4073 tested probands have a phenotype compatible with our DD gene list. Although only 42% of the participants were recorded as having “Neurology and neurodevelopmental disorders,” 3259 of 4037 (78%) belonged to a group of recently defined “DDD-like” individuals (Huang QQ, Wigdor EM, Campbell P, et al. Dissecting the contribution of common variants to risk of rare neuro-developmental conditions. medRxiv. Published online March 6, 2024.03.05.24303772. https://doi.org/10.1101/2024.03.05.24303772), whose phenotypes are broadly in keeping with the phenotypes of participants recruited to the DDD study. Of the 6 individuals with likely or candidate diagnoses, 3 were classed as “DDD-like” (*PAH, LAMA2*, and *IGHMBP2*), whereas the 3 most confident diagnoses were not (*NPHP3, GAA*, and *PKHD1*). Second, we focused our search for noncoding variants in regions within (introns and UTRs) or with clear links to genes (directly upstream, or coaccessible with the upstream region in sci-ATAC-seq data from fetal brain), defined using a single representative transcript per gene. This approach will exclude many regulatory elements, including more distal enhancer regions, although arguably captures the regions most likely to contain variants of large effect. Third, we focused on single-nucleotide variations and small indels and therefore will have missed noncoding structural variants (such as the most likely second hit in *ALMS1*, which was an exonic deletion). Fourth, it was necessary to perform very strict filtering of candidate noncoding variants to reduce the number for clinical review because a substantial proportion of our proband-variant pairs (~36%) had at least 1 noncoding variant in trans. Fifth, it is likely that some individuals with noncoding second hits will have already been diagnosed by GEL and hence not have been included in our initial cohort. These would include probands with splice region variants that were flagged as tier 1 or 2 or individuals for which the recruiting center looked at tier 3 variants (potentially because local testing flagged a single hit in a candidate recessive gene). Finally, because of the difficulty of recontacting recruiting clinicians through the GEL framework, we limited our follow-up efforts to only individuals for whom our initial clinical review suggested a variant pair as the “probable” cause of the reported phenotype. It is likely that some of the variants/genes classified as “possible” are also bona fide diagnoses, especially given the phenotypic information within GEL is often incomplete.

We used a comprehensive annotation and filtering approach across gene-proximal regulatory elements, annotating variants that affect known regulatory elements (miRNA binding, polyadenylation, translational control elements, etc) and/or that are flagged as deleterious by a range of in silico predictors (SpliceAI, CADD, and PhyloP). Despite this thorough approach, identifying disease-causing noncoding variants remains very difficult. Indeed, in 2 cases (*GAA* and *ALMS1*), our initial filtering missed what were ultimately deemed to be the most likely “second hits.” We are still lacking knowledge of the “regulatory code” and tools to effectively filter non-coding region variants. Our stringent region-specific filtering approach could likely be improved as knowledge of noncoding region variants and their impacts in disease continues to evolve.

A large proportion of our identified noncoding second hits are intronic. This is expected, given that the vast majority of the search-space per gene (~90%) is intronic. It is also relevant to note that recessive DD genes on average have much shorter 5′ UTRs than the average across all genes (and, indeed, their dominant counterparts), reflecting the lower importance of translational regulation in these genes.^44^ We would therefore not expect to find many high-impact 5′ UTR variants across our gene set.

Our analyses, along with prior reports, suggest that the *GAA* 5′ UTR c.-32-13T>G splice-altering variant is a more likely damaging second hit than the initially identified promoter variant in cis. This 5′ UTR variant was not picked up in our filtering pipeline because it was over our allele frequency threshold. This underscores the potential for hypomorphic variants, ie, those that are not complete loss of function, to be found at higher frequencies in the population. Using RNA-seq, we confirmed the previously reported skipping of exon 3, which contains the start codon, and found evidence of full intron retention that has not previously been reported, likely because of differences in methodology. Prior validations had utilized RT-PCR or minigene assays, neither of which would be expected to detect this intron retention event, because of the much larger size of the amplicon or the lack of native context used. RNA-seq of other probands with the variant, particularly long read sequencing, would help clarify the exact nature of the aberrant transcripts generated. Larger-scale missplicing events, such as full intron retention and multi-exon skipping, have been reported to be less frequent than single-exon skipping or cryptic splice site usage.^45^ With increasing use of RNAseq, more of these larger scale events may be detected, potentially revealing them to be more common than previously appreciated. This may necessitate the reclassification of variants previously believed not to affect splicing but whose effects were not adequately captured by previous methodologies.

Our results have important implications for genetic testing guidelines and consideration of the strength of evidence assigned to variants found in trans with pathogenic coding variants (ie, activation of PM3 in the ACMG/AMP framework^46^). Despite the increase in search-space when including noncoding regulatory region variants, with a careful filtering approach the number of candidate non-coding second hits can be kept to a manageable level, and hence PM3 can still be applied at a moderate strength.^47^

In summary, we have developed a systematic approach to identify noncoding second hits in recessive genes, highlighting how damaging noncoding variants can be annotated to find new diagnoses for undiagnosed DDs but also the challenges in doing this effectively. Through this work, we conclude that this mechanism is unlikely to account for a large proportion of missing DD diagnoses. Future work should couple RNAseq with genome sequencing to identify additional pathogenic noncoding variants, but it should also consider other potential explanations for undiagnosed patients, such as coding variants in novel genes,^2^ as well as oligogenic and polygenic contributions.^48^

## Supplementary Material

Supplementary Material

## Figures and Tables

**Table 1:** Candidate coding/noncoding variant pairs

Gene; HGNC ID	Phenotype		Coding Variant HGVS; chr:pos:ref:alt	Noncoding Region Variant					
	Normalized Specific Disease	Abstracted Selected HPO Terms		Details HGVS; chr:pos:ref:alt	gnomAD FAF	GEL AF	SpliceAI	PhyloP	CADD
*GAA;* HGNC:4065	Limb girdle muscular dystrophy	Abnormality of the calf musculature; muscular dystrophy; respiratory insufficiency; Abnormality of the eye; progressive muscle weakness	NC_000017.11:g.80118288G>A; ENST00000302262.8:c. 2577G>A; p.(Trp859Ter); chr17:80118288:G:A Nonsense	NC_000017.11:g.80101399C>G; NG_029761.1:g.69768C>G; chr17:80101399:C:G Core Promoter	2.75 × 10^−3^	3.17 × 10^−3^	NA	−0.19	5.88
*NPHP3;* HGNC:7907	Proteinuric renal disease	Abnormal renal corpuscle morphology; abnormal liver morphology; abnormal urine metabolite level	NC_000003.12:g.132691199G>A; ENST00000337331.10: c.2563C>T; p.(Gln855Ter); chr3:132691199:G:A Nonsense	NC_000003.12:g.132684549C>T; ENST00000337331.10:c.3570 +5G>A;chr3:132684549:C:T Intronic	5.14 × 10^−6^	8.95 × 10^−5^	0.04	6.15	21.0
*ALMS1;* HGNC:428	Cone dysfunction syndrome	Abnormal visual electrophysiology; Abnormal eye physiology; Abnormal retinal morphology; Abnormality of vision	NC_000002.12:g.73572649del; ENST00000613296.6:c. 10772del; p.(Thr3591LysfsTer6); chr2:73572648:AC:A Frameshift	NC_000002.12:g.73573562G>A; ENST00000613296.6:c.11547 +138G>A; chr2:73573562:G:A Intronic	1.72 × 10^−3^	1.73 × 10^−3^	0.01	3.84	20.2
*LAMA2;* HGNC:6482	Congenital myopathy	Abnormal skeletal muscle morphology; muscle weakness; abnormal muscle physiology; abnormal joint physiology	NC_000006.12:g.129316089C>T; ENST00000421865.3:c.3976C>T; p.(Arg1326Ter); chr6:129316089:C:T Nonsense	NC_000006.12:g.129475370dup; ENST00000421865.3:c.7440 -20dup; chr6:129475360:G:GT Intronic	4.71 × 10^−4^	5.88 × 10^−4^	0.10	NA	8.48
*IGHMBP2;* HGNC:5542	Charcot-Marie- Tooth disease	Peripheral axonal degeneration	NC_000011.10:g.68936909del; ENST00000255078.8: c.2429del; p.(Pro810LeufsTer21); chr11:68936904:GC:G Frameshift	NC_000011.10:g.68929807G>A; ENST00000255078.8:c.1235 +450G>A; chr11:68929807:G:A Intronic	9.51 × 10^−5^	1.66 × 10^−4^	0.12	−1.91	0.21
*PKHD1;* HGNC:9016	Cystic kidney disease	Abnormality of urine homeostasis; abnormality of urethra; abnormality of the kidney; abnormal renal morphology	NC_000006.12:g.52028249G>A; ENST00000371117.8: c.3467C>T; p.(Ser1156Leu); chr6:52028249:G:A Missense	NC_000006.12:g. 51882440T>C; ENST00000371117.8:c.7350 +653A>G; chr6:51882440:T: C Intronic	0.00	7.68 × 10^−5^	0.95	−0.30	8.22
PAH; HGNC:8582	Undiagnosed metabolic disorders	Abnormality of metabolism/ homeostasis; tremor; abnormality of bone mineral density	NC_000012.12:g.102844359G>C; ENST00000553106.6:c.1042C>G; p.(Leu348Val); chr12:102844359:G:C Missense	NC_000012.12:g.102843790C>T; ENST00000553106.6: c.1066-11G>A; chr12:102843790:C:T Intronic	3.74 × 10^−4^	3.84 × 10^−4^	0.98	0.88	23.5

Shown are variant details, selected annotations, and phenotypic data relating to the proband. All chromosome coordinates related to GRCh38. *AF*, allele frequency; *FAF*, gnomAD v3.0 filtering AF; *HPO*, Human Phenotype Ontology.

## Data Availability

Data were analyzed within the Genomics England secure research environment. All shareable data were exported from the research environment with approval and are included as supplementary tables. Because of its large size, Supplemental Table 2 is hosted on GitHub: https://github.com/Computational-Rare-Disease-Genomics-WHG/non-coding_second_hits.
