# Supplementary material for: Noncoding variants are a rare cause of recessive developmental disorders in trans with coding variants
Source: Genet Med. Author manuscript; Available in PMC 2025 Jun 24. (PMC7617801; doi:10.1016/j.gim.2024.101249)
Supplement: Supplementary Material [file EMS206547-supplement-Supplementary_Material.zip › 1-s2.0-S1098360024001837-mmc1.pdf]

# Supplementary Data

## Supplementary Table legends

**Supplementary Table 1**: List of DDG2P recessive genes and MANE Select v1.0 transcripts

**Supplementary Table 2**: List of annotated non-coding regions for each DDG2P recessive gene.

**Supplementary Table 3**: Overview of the non-coding search space. Shown is the total search-space across all transcripts corresponding to each region type. Note, a base could be counted more than once if it has different annotations for different genes. The mean, min and max are calculated for the number of genes with at least a single base assigned to that region.

**Supplementary Table 4**: rMATS differential splicing results for proband with GAA variants. Information on the differential splicing event associated with the GAA UTR variant, including coordinates of exons, counts of reads supporting the skipping and inclusion events, FDR corrected p-value and inclusion level difference between the proband and the controls.

## Supplementary Figures

**Supplementary Figure 1: Listed 'normalised disease group' for the initial set of 4,073 undiagnosed probands.** 1,711 (42%) have "Neurology and neurodevelopmental disorders" as a listed normalised disease group. Due to Genomics England rules, disease groups containing fewer than five probands have been removed.

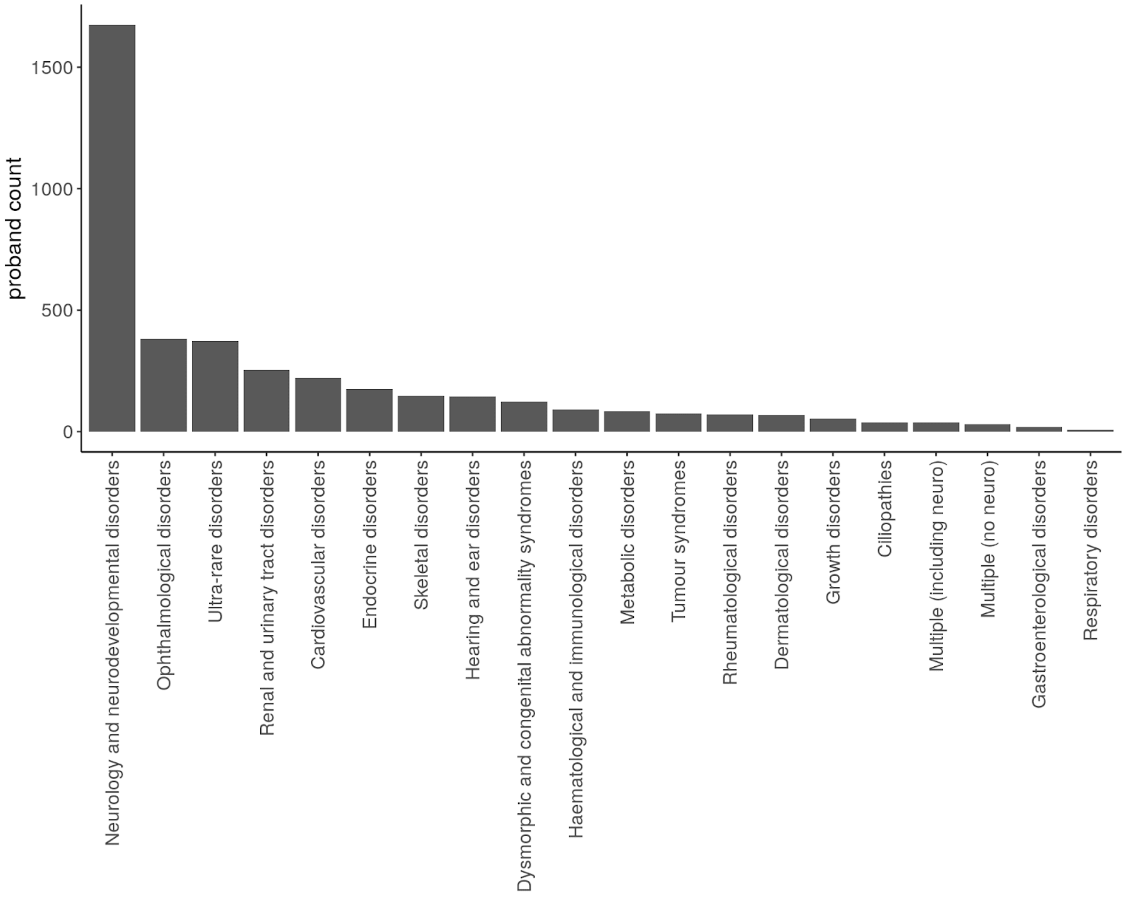

**Supplementary Figure 2: RT-PCR investigations of *LAMA2* and *IGHMBP2* potential splicing variants.** A) Schematic diagram of *LAMA2* RT-PCR (red curves indicate splicing junctions, green arrows primer locations). B) Sequence of forward and reverse primers used in *LAMA2* RT-PCR. C) Gel electrophoresis showing single band generated by RT-PCR of both the case and control. D) Electropherogram showing Sanger sequencing data from the case and control, with both case and control displaying skipping of exon 53. E) Schematic diagram of *IGHMBP2* RT-PCR (red curves indicate splicing junctions, green arrows primer locations. F) Sequence of forward and reverse primers used in *IGHMBP2* RT-PCR. G) Gel electrophoresis showing consistent patterns of splicing between the case and controls.

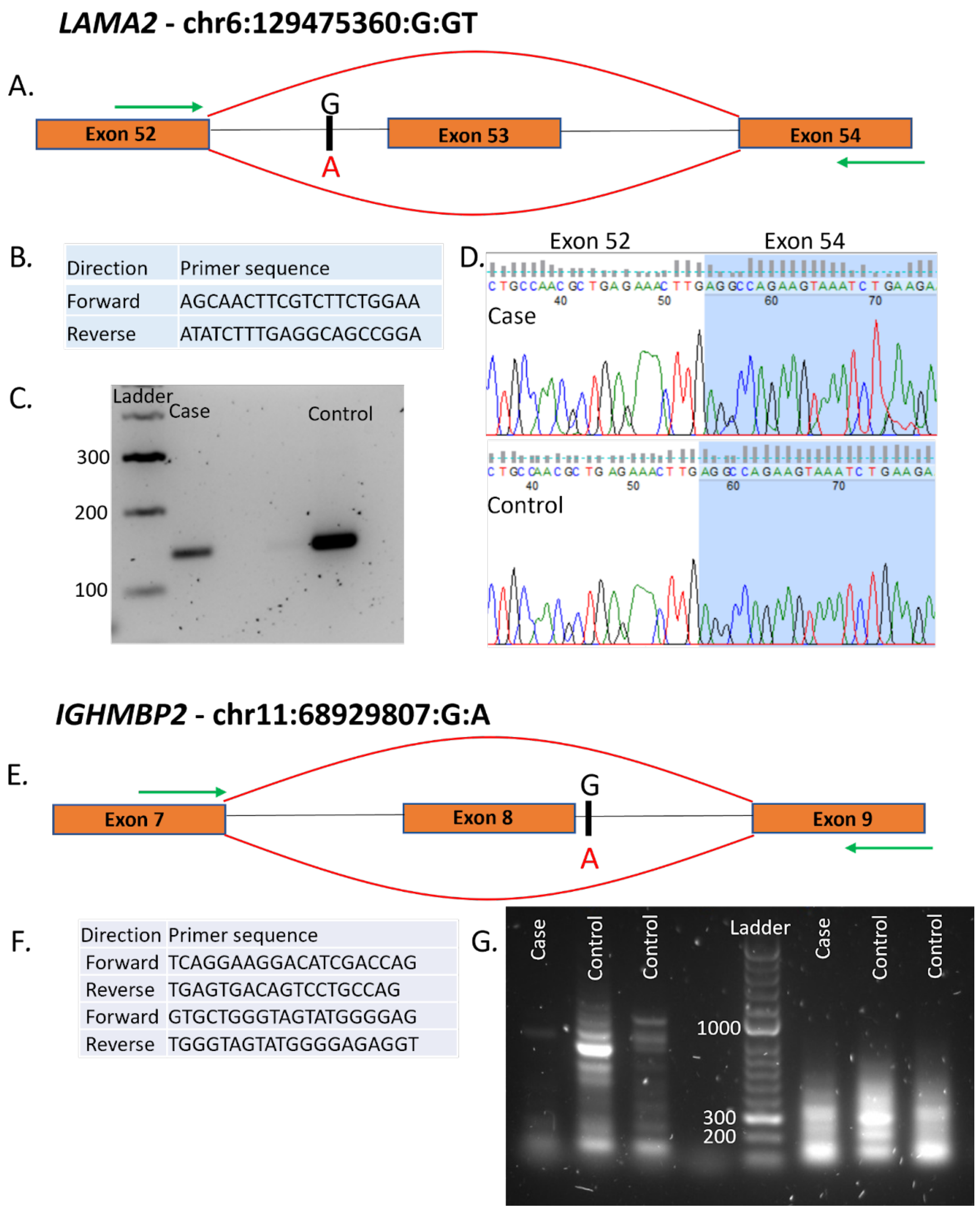


B.

| Direction | Primer sequence |
|---|---|
| Forward | AGCAACTTCGTCTTCTGGAA |
| Reverse | ATATCTTTGAGGCAGCCGGA |

F.

| Direction | Primer sequence |
|---|---|
| Forward | TCAGGAAGGACATCGACCAG |
| Reverse | TGAGTGACAGTCCTGCCAG |
| Forward | GTGCTGGGTAGTATGGGGAG |
| Reverse | TGGGTAGTATGGGGAGAGGT |
